# Persistent Leatherback Turtle Migrations Present Opportunities for Conservation 

**DOI:** 10.1371/journal.pbio.0060171

**Published:** 2008-07-15

**Authors:** George L Shillinger, Daniel M Palacios, Helen Bailey, Steven J Bograd, Alan M Swithenbank, Philippe Gaspar, Bryan P Wallace, James R Spotila, Frank V Paladino, Rotney Piedra, Scott A Eckert, Barbara A Block

**Affiliations:** 1 Hopkins Marine Station, Stanford University, Pacific Grove, California, United States of America; 2 Joint Institute for Marine and Atmospheric Research, Honolulu, Hawaii, United States of America; 3 NOAA/NMFS/SWFSC/Environmental Research Division, Pacific Grove, California, United States of America; 4 Collecte Localisation Satellites, Direction Océanographie Spatiale, Ramonville, France; 5 Center for Applied Biodiversity Science, Conservation International, Arlington, Virginia, United States of America; 6 Department of Bioscience and Biotechnology, Drexel University, Philadelphia, Pennsylvania, United States of America; 7 Department of Biology, Indiana-Purdue University, Fort Wayne, Indiana, United States of America; 8 Parque Nacional Marino Las Baulas, Ministerio de Ambiente y Energía, San José, Costa Rica; 9 Wider Caribbean Sea Turtle Conservation Network, Duke University Marine Laboratory, Beaufort, North Carolina, United States of America; Imperial College, United Kingdom

## Abstract

Effective transboundary conservation of highly migratory marine animals requires international management cooperation as well as clear scientific information about habitat use by these species. Populations of leatherback turtles (Dermochelys coriacea) in the eastern Pacific have declined by >90% during the past two decades, primarily due to unsustainable egg harvest and fisheries bycatch mortality. While research and conservation efforts on nesting beaches are ongoing, relatively little is known about this population of leatherbacks' oceanic habitat use and migration pathways. We present the largest multi-year (2004–2005, 2005–2006, and 2007) satellite tracking dataset (12,095 cumulative satellite tracking days) collected for leatherback turtles. Forty-six females were electronically tagged during three field seasons at Playa Grande, Costa Rica, the largest extant nesting colony in the eastern Pacific. After completing nesting, the turtles headed southward, traversing the dynamic equatorial currents with rapid, directed movements. In contrast to the highly varied dispersal patterns seen in many other sea turtle populations, leatherbacks from Playa Grande traveled within a persistent migration corridor from Costa Rica, past the equator, and into the South Pacific Gyre, a vast, low-energy, low-productivity region. We describe the predictable effects of ocean currents on a leatherback migration corridor and characterize long-distance movements by the turtles in the eastern South Pacific. These data from high seas habitats will also elucidate potential areas for mitigating fisheries bycatch interactions. These findings directly inform existing multinational conservation frameworks and provide immediate regions in the migration corridor where conservation can be implemented. We identify high seas locations for focusing future conservation efforts within the leatherback dispersal zone in the South Pacific Gyre.

## Author Summary

Highly migratory marine animals routinely cross international borders during extensive migrations over thousands of kilometers, thus requiring conservation strategies with information about habitat use and movement patterns. Critically endangered leatherback turtles (Dermochelys coriacea) in the eastern Pacific have suffered a severe population decline in recent years. In this study, we present the largest multi-year satellite tracking data set for leatherback turtles (*n* = 46 turtles, 12,095 days) to describe the migrations, habitats, and dispersal of female leatherbacks tagged at Playa Grande, Costa Rica. Leatherbacks followed a migration corridor southward from Costa Rica into the South Pacific Gyre in each year of our study. In the equatorial region, leatherbacks experienced strong ocean currents that influenced the direction of their movements; leatherbacks responded to current deflection with rapid, directed movements to maintain their southward heading. After passing through this equatorial current field, turtles dispersed broadly within a low-energy, low-productivity region of the South Pacific. Our analyses revealed that ocean currents shaped the migration corridor and influenced the scope of turtle dispersal in the South Pacific—results that provide a biological rationale for the development of multi-scale conservation strategies. These strategies could involve improved and enhanced monitoring of leatherback–fisheries interactions as well as dynamic time-area fisheries closures and protected area designations within the high seas of the South Pacific.

## Abstract 3

Satellite tracking data from female leatherback turtles reveal their migration routes in the eastern Pacific and demonstrate how oceanic currents shape their migration corridors, providing a biological basis for conservation strategies.

## Introduction

Leatherback turtles (Dermochelys coriacea) in the eastern Pacific (EP) have exhibited population declines of up to 90% during the past two decades [1,2]. These declines have been driven by a number of factors, including incidental mortality in fisheries, loss of nesting habitats, and unsustainable egg harvest [1,3]. Of the extant leatherback nesting beaches in the EP, Playa Grande in Parque Nacional Marino Las Baulas (PNMB), Costa Rica, supports the largest nesting colony [1]. After the nesting period (approximately 60 d), EP leatherbacks perform long-distance migrations from breeding areas to feeding areas, where they remain for 2 to 7 y [4]. Therefore, while protection of nesting habitat is important to enhance recruitment into the population, an improved understanding of the at-sea distribution and movements of EP leatherbacks is vital to ensuring their long-term survival. In particular, long-range tracking studies using electronic tags can inform conservation efforts by identifying high-use areas for leatherbacks in time and space, as well as environmental influences on leatherback behavior [5].

Leatherback turtles globally undertake long-distance migrations over thousands of kilometers [6–14]. Morreale et al. [6] first described the movements of EP leatherbacks from the tracks of eight turtles (durations 3–87 d) and identified a persistent southbound migration corridor from PNMB toward the Galápagos Islands. Additional tagging efforts at a nesting beach in Mexiquillo, México, about 965 km north of Costa Rica, revealed that leatherbacks traveled routes that shared the same directional heading and general high seas habitats in the eastern South Pacific as those traveled by Costa Rican turtles [7]. In contrast, leatherbacks from other populations demonstrate inter-individual behavioral variation with respect to post-nesting migration routes [8–10,13,14]. The apparent persistence of the EP leatherback migration pattern provides a unique opportunity to generate a cohesive conservation management approach for this endangered population.

Conservation of highly migratory marine species requires international cooperation for implementation of transboundary management strategies. Specifically, information on movements and distributions of large marine predators collected by electronic tracking devices can provide guidance to the development of national and multinational fisheries management strategies and bycatch mitigation efforts, as well as support related policy efforts [15]. One such framework is the Eastern Tropical Pacific Seascape (ETPS) initiative [16], which is a multinational coordination of marine resource management within the combined exclusive economic zones of Costa Rica, Panama, Colombia, and Ecuador. The ETPS is an area that is home to several marine protected areas (MPAs) (e.g., PNMB) and World Heritage sites (e.g., Cocos Island, Coiba Island National Park, Malpelo Island, Galápagos Islands and Marine Reserve). Thus, the ETPS represents a framework through which habitat use and movement data for migratory animals, such as leatherbacks, can be translated into tangible management actions.

Here we present the largest multi-year tracking data set collected for this species, based on 46 individuals satellite-tagged during 2004–2007 at PNMB. Our approach is consistent with a recent review [17], which emphasized the importance of tracking large sample sizes and an interdisciplinary approach integrating oceanographic cues with behavior. These data enabled us to (1) describe the distribution and horizontal movements of leatherbacks in the EP, (2) examine the influence of oceanic currents on leatherback migrations, (3) assess leatherback high-use habitats, (4) confirm and elucidate a leatherback migration corridor from the nesting beach to 5 °S, and (5) describe leatherback movements beyond 10 °S into the South Pacific. In addition, these data identify critical areas for directed conservation efforts to ensure the survival of this species in the EP.

## Results

**Figure 1:**
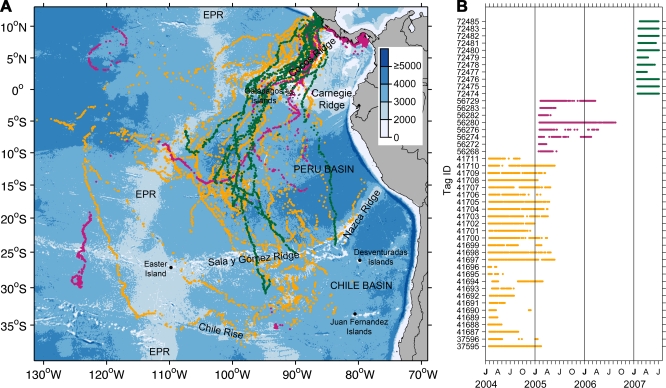
Map and Timeline of Leatherback Sea Turtle Tracking Data (A) Satellite transmission positions for 46 leatherback turtles from 2004 (*n* = 27, orange), 2005 (*n* = 8, purple), and 2007 (*n* = 11, green), tagged at Playa Grande, Costa Rica, overlaid on bathymetry (in m). Prominent bathymetric features and island groups are labeled (EPR = East Pacific Rise). (B) Timeline of satellite transmissions for each tag (tag ID is the ARGOS-assigned transmitter number).

We tagged 46 female leatherback turtles during oviposition, resulting in 12,095 tracking days spanning 21 January 2004–5 July 2007, with a mean track duration of 263 d, a distance of 8,070 km, and a travel speed of 37.7 km d^−1^ (Table 1). Movements by cohorts from a given year displayed cohesion, even though initiation of the post-nesting migration among individuals differed by up to several weeks (Figure 1). Only one individual tagged in 2005 (tag ID 56280) remained in coastal waters off Costa Rica and Panama for the entire tag duration (Figure 1A).

**Table 1:**
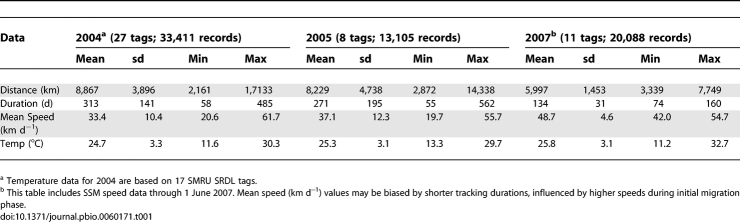
Tracking Data from 46 Satellite-Linked Tags Deployed on Leatherback Turtles on Playa Grande, Costa Rica, 2004–-2007

**Figure 2:**
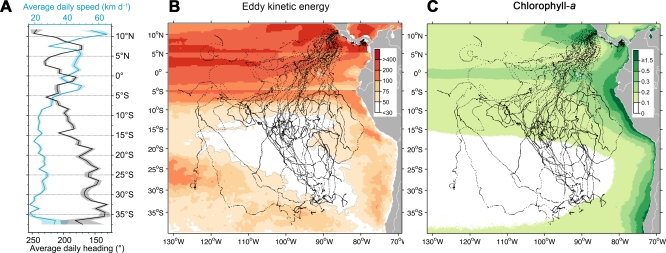
Large-Scale Oceanographic Characteristics and Leatherback Movements in the EP Turtle median daily positions (black dots) generated with state-space model interpolation [21], overlaid on long-term mean. (A) Turtle median daily speed (blue line) and heading (black line), with corresponding standard-error envelopes, averaged in one-degree latitudinal bins. (B) *EKE* (in cm^2^ s^−2^). (C) Near-surface CHL concentration (in mg m^−3^).

Upon completion of nesting activity, leatherbacks embarked on rapid (42.9 km d^−1^, standard deviation (sd) = 27.7 km d^−1^) directed southward migrations through the equatorial region. Once south of 5 °S, the turtles dispersed throughout the South Pacific Gyre following slower (23.8 km d^−1^, sd = 16 km d^−1^), meandering paths, and remained there through the duration of the tracking period (Figure 2A–2C). Across their migrations, turtles experienced a wide range of surface temperatures (11.2–32.7 °C, mean = 25.2 °C, sd = 3.2 °C; Table 1). They encountered areas of high–eddy kinetic energy (*EKE*) in the equatorial region (>100 cm^2^s^−2^), and areas of very low *EKE* (<50 cm^2^s^−2^) in the dispersal region (Figure 2B). Likewise, chlorophyll-*a* (CHL) concentrations were highest in the equatorial region (>0.3 mg m^−3^), and lowest in the South Pacific Gyre (<0.1 mg m^−3^) (Figure 2C). Swimming speed was significantly higher in areas of high CHL and vice-versa (linear regression: β = 0.964 ± 0.057, *F*_1,9577_ = 281, *p* < 0.001, *r* ^2^ = 0.029), although the association between these two variables was weak.

**Figure 3:**
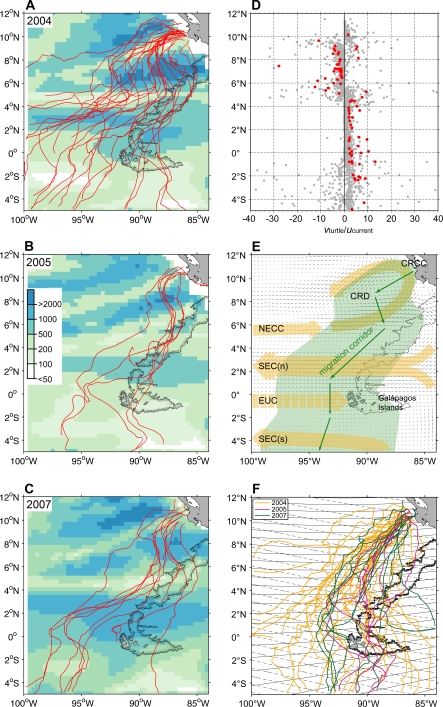
Leatherback Movements in Relation to Ocean Currents in the Migration Corridor Region (A–C) Turtle tracks overlaid on *MKE* (in cm^2^ s^−2^) for February–April periods for 2004, 2005, and 2007. Stippling shows the 2000-m isobath highlighting the Cocos Ridge. (D) Ratio of turtle meridional velocity to current zonal velocity in the migration corridor region. Points corresponding to turtle meridional velocities faster than −70 km d^−1^ are colored in red. (E) Schematic of turtle migration corridor through the equatorial current system (current abbreviations are given in the text), based on the 75% home-range utilization distribution contour. (F) Current corrected turtle tracks from 2004 (orange), 2005 (green), and 2007 (purple), overlaid on contours of magnetic force (solid thin black lines) and magnetic inclination (dashed thin black lines). The force field has an intensity ranging from 38,314 nT in the north to 27,798 nT in the south, and contours are drawn every 420 nT. The inclination field ranges from 1.6° and 43.1°, and contours are drawn every 1.7°.

Ocean current energetics had a major impact on the turtles' migration route. Between latitudes 12 °N and 5 °S, southbound turtles negotiated the strong alternating eastward-westward flows of the equatorial current system, whose strength can be of comparable magnitude to turtle travel speeds (Text S1 and Figure S1). Turtles initially moved rapidly WSW (mean speed = 63.8 km d^−1^, sd = 31.8 km d^−1^; mean heading = 247°, sd = 40°) through a narrow zone of low mean kinetic energy (*MKE*) near 10 °N between the southern edge of the Costa Rica Dome (CRD) and the Costa Rica Coastal Current (CRCC) (Figure 3A–3C, Text S1, and Figure S1). They then crossed the energetic flow along the southern edge of the CRD between 8 °N and 6 °N on a SE heading (mean speed = 49.9 km d^−1^, sd = 27.8 km d^−1^; mean heading = 173°, sd = 42°). Once outside the CRD, turtles turned WSW (mean speed = 50.2 km d^−1^, sd = 26.4 km d^−1^; mean heading = 225°, sd = 43°) over another area of low *MKE* near 4 °N and continued rapidly on this course aided by the westward-flowing northern branch of the South Equatorial Current (SEC) near 3 °N. Between 1 °N and 2 °S, turtles turned southward (mean speed = 41.7 km d^−1^, sd = 22.9 km d^−1^; mean heading = 186°, sd = 46°), as they rapidly crossed the Equatorial Undercurrent (EUC) by again increasing their southward speed, even while being advected eastward by the EUC (Figure 3A–3C). A final SW turn (mean speed = 43.5 km d^−1^, sd = 17.8 km d^−1^; mean heading = 196°, sd = 37°) occurred as the turtles crossed the much weaker southern branch of the westward SEC between 3 °S and 5 °S.

Examination of the ratio of turtle meridional velocity to current zonal velocity in the 12 °N–5 °S region revealed that in areas of strong currents, the turtles responded by increasing their southward velocity regardless of flow direction (i.e., ratios consistently close to zero in the 8 °N–6 °N and 4 °N–1 °N latitudinal bands; Figure 3D). After the effect of the currents was removed (Figure 3F), the tracks appeared much straighter for all years, showing a consistent SSW heading between 12 °N and 1 °N (mean = 193°, sd = 30°) and a southward heading afterward. The contours of geomagnetic force were generally oriented NE-SW while the contours of geomagnetic inclination were generally oriented NW-SE, forming a grid pattern in the 12 °N–5 °S region (Figure 3F). The current-corrected tracks generally crossed these magnetic gradients from north to south (Figure 3F).

## Discussion

### Elucidation of the EP Leatherback Migration Corridor: The Influence of Ocean Currents

This multi-year dataset confirmed the existence of a persistent migration corridor for leatherbacks spanning from the Pacific coast of Central America, across the equator and into the South Pacific. The turtles traveled along a predominantly southwesterly heading, which was strongly influenced by ocean currents. An earlier telemetry study hypothesized a leatherback migration corridor between Costa Rica and Galápagos that could be influenced by environmental factors such as ocean fronts, bathymetric features, currents, or geomagnetic cues [6]. We examined each of these hypotheses with our larger sample size, and found no relationship between the southward turtle movements and the most prominent frontal feature in the region, the Equatorial Front, which runs east to west just north of the equator (Text S1). We also found no consistent association between leatherback tracks and the Cocos Ridge, the dominant bathymetric feature in the region, even after current correction (mean turtle heading = 193° versus 224° if they had followed the orientation of the Ridge). Instead, the turtles' movements over the Cocos Ridge were correlated with the current strength of the southern edge of the CRD, which deflected them over portions of the Ridge each year (Figure 3A–3C). Once the influence of currents was removed, it was apparent that the turns observed in the tracks in the corridor region (12 °N–5 °S) were current-induced. For these reasons, we conclude that navigation through the complex and highly energetic equatorial region supports the existence of a compass sense, possibly guided by the geomagnetic map (Figure 3F) formed by the force and inclination fields in the region, as has been documented for sea turtles in other parts of the world [18].

Our results demonstrate that leatherbacks responded to strong zonal currents by increasing their southward speed, probably to maintain their SSW headings and to avoid being pushed too far eastward or westward from their destination in the South Pacific Gyre. Inter-annual variability in current location and strength was a major force shaping the turtles' migration routes. In each year, the migration corridor was initially constrained to the zone of lowest *MKE* associated with the center of the CRD (Figure 3A–3C) and, ultimately, the breadth of their dispersal within the South Pacific Gyre (Figure 2B) was determined by the strength of the equatorial currents through which they migrated. This was particularly evident in 2005, when the currents were weaker, and in 2007, with stronger currents (Figure 3B and 3C). This interaction between post-nesting EP leatherbacks and currents contrasts with that of South African leatherbacks [19], whose variable long-distance movements suggest passive drift with prevailing currents in the Southwest Indian Ocean.

Leatherbacks moved rapidly through the productive equatorial region [20,21] and then dispersed in the most oligotrophic region of the Pacific Ocean [22]. The slow, meandering movements by the turtles in the South Pacific Gyre suggest that post-nesting female leatherbacks probably migrate there to forage. Leatherback turtles do not feed directly on phytoplankton but on large gelatinous zooplankton [23], and despite its low phytoplanktonic biomass, the South Pacific Gyre ecosystem sustains an ample mesozooplanktonic forage base and a substantial longline tuna fishery [24,25]. Therefore, we suggest that following the energetic demands of egg production, it may be more efficient for post-nesting EP leatherbacks to forage in an oceanographically quiescent region within which high water clarity could enhance prey detection [26] while requiring minimal swimming effort.

A further possible explanation for the consistency in migration routes followed by EP leatherbacks is that present-day migration patterns do not reflect the historic diversity of migration strategies, such as that observed in other leatherback populations [13,14,19,27]. The leatherback tracks presented here, which document the first 12–18 mo of the entire ∼4-y remigration interval, suggest that post-nesting EP turtles almost exclusively occupy oceanic areas. Eckert and Sarti [7] also tracked EP leatherbacks to oceanic areas, but a few of these turtles moved into coastal areas off South America. A single turtle in this study (tag ID 56280, tagged during 2005) occupied exclusively nearshore foraging habitats along the coast of Central America throughout the entirety of its tracking duration (562 d). Previous reports have indicated substantial leatherback bycatch in nearshore fisheries in the EP, specifically in swordfish driftnets off Chile and Peru [7,28,29], and leatherbacks continue to interact with fisheries in Peruvian [30] and Chilean waters [31]. Given that coastal areas in the EP represent highly productive areas when compared with oceanic areas, Saba et al. [32] hypothesized that this bycatch could have essentially extirpated a “coastal” migratory phenotype in this population. Sustained tracking efforts on the EP population, including continuous tracking studies on previously tagged remigrant turtles, and tagging of turtles in foraging habitats are necessary to test this hypothesis.

### Conservation Implications of EP Leatherback Tracking Data

Characterization of spatio-temporal habitat use is a fundamental element of effective biodiversity conservation management strategies. Our results have enabled us to define two high-use areas for leatherback turtles in the EP: (1) an oceanic post-nesting migration corridor shaped by currents, and (2) and putative foraging grounds in the South Pacific Gyre. The data provide compelling new strategies for conservation of Pacific leatherbacks (Box 1), which could also benefit other marine species.

**Box 1. Conservation Recommendations from EP Leatherback Tracking Data**

1. Implement specific strategies for leatherback protection within existing MPAs and conservation initiatives in the EP.

2. Improve collection of fisheries-dependent information to characterize leatherback-fisheries interactions in the EP.

3. Increase understanding of multi-species movements and high-use areas through the use of fisheries-independent data (i.e. electronic tagging).

4. Apply recommendations 1–3 above for planning of dynamic time-area closures and/or appropriate gear modifications to reduce leatherback-fisheries interactions within the following high-use areas:

• EP leatherback high-use area #1: Post-nesting migration corridor spanning an open-ocean region from the Pacific coast of northwest Costa Rica (approximately 12°N) to approximately 5°S, within which turtles are seasonally concentrated during the period of February-April.

• EP leatherback high-use area #2: Defined by the predictable association of turtles during their dispersal phase within putative foraging habitats in the region of current motions (i.e. eddy kinetic energy) in the South Pacific Gyre.

**Figure 4:**
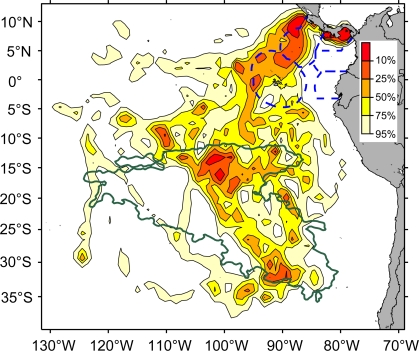
Combined Utilization Distribution by EP Leatherback Turtles from all Tracking Data EP leatherback turtle home-range utilization distribution for all years combined (2004, 2005, and 2007). Boundaries of the ETPS, corresponding to the exclusive economic zones of Costa Rica, Panama, Ecuador, and Colombia, are shown as dashed blue lines. The green polygon comprises the region with the lowest climatological *EKE* (≤30 cm^2^ s^−2^) in the South Pacific Gyre.

First, we encourage enhanced regional and international cooperation in management of leatherbacks and their migration corridor occurring within existing MPAs (i.e., PNMB, Cocos Island, Coiba Island, Galápagos Islands) and conservation initiatives (i.e., ETPS). Because much of the leatherback dispersal region occurs within international waters, multinational organizations and policy instruments (i.e., Inter-American Convention for the Protection and Conservation of Sea Turtles, Inter-American Tropical Tuna Convention, Convention on Highly Migratory Species, Comisión Permanente del Pacífico Sur, United Nations Convention on the Law of the Sea, and South Pacific Regional Fisheries Management Organization) should be leveraged to achieve turtle management and conservation outcomes on the high seas. The leatherback migration corridor, which occurs during a period of a few months (February–April, Figure 3H), is largely contained within the boundaries of the ETPS, which also includes PNMB and the coastal areas used by one of the tagged turtles. This affords an opportunity for each of the governments involved in these initiatives to actively participate in the spatio-temporal management of leatherbacks as they occupy the network of MPAs in this region (Figure 4).

Second, we strongly recommend enhanced and increased collection of fisheries-dependent bycatch data and fisheries-independent habitat use data throughout the EP. In particular, expanded and improved observer coverage in EP fisheries would be an important step to characterize leatherback interactions with fisheries operations in the EP, because currently available information on leatherback bycatch is inconsistently collected for most fisheries. Collection of fisheries bycatch data for leatherbacks is critical to evaluating relative effects of distinct fisheries on leatherback mortality. Leatherback interactions with small-scale fisheries (i.e., artisanal, traditional, subsistence) may be especially critical in coastal habitats where extremely high sea turtle bycatch rates have been observed [7,33].

For fisheries-independent information, further electronic tagging efforts on EP leatherbacks and other highly migratory marine species that share similar high seas habitats and face common human threats would improve effectiveness of adaptive conservation schemes. Our data represent only the initial segment of the entire nonreproductive period for female leatherbacks, leaving much of their at-sea behavior and habitat use unexplored. Therefore, a priority for future leatherback tagging studies should be to focus on foraging ground behavior and movements throughout the entire nonreproductive period.

Third, improved data on fisheries bycatch and leatherback habitat use in the EP as outlined above would inform planning of dynamic time-area closures and/or appropriate gear modifications intended to reduce turtle interactions with fisheries. A current illustration of this approach is in the USA California/Oregon-based drift-gillnet and longline fisheries, where a time-area closure was implemented based on temporal and spatial patterns of leatherback distributions; leatherback bycatch was reduced to zero following implementation of this measure [34]. This tracking study defines at least two regions within the Pacific where strategic time-area closures could be a useful management tool for protecting leatherbacks within high-use habitats. The first area is the post-nesting migration corridor spanning an open-ocean region from the Pacific coast of northwest Costa Rica (approximately 12 °N) to approximately 5 °S, within which turtles are seasonally concentrated, in predictable patterns, during the period of February–April. The second high-use area is defined by the predictable association of turtles during their dispersal phase (through putative foraging habitats) with the low *EKE* region of the South Pacific Gyre. Time-area closures could be applied to protect post-nesting turtles when they are seasonally concentrated during migration (i.e., while moving through the ETPS) and within international waters on the high seas during foraging periods within low *EKE* regions of the South Pacific Gyre. Another specific opportunity to manage turtles during dispersal occurs when they pass through the oceanic island territories of Chile (i.e., Easter Island, Juan Fernandez Islands, and the Desventuras Islands). Management actions within each of the above regions should be based upon spatio-temporal overlap of the leatherback high-use areas and areas of high bycatch. In addition to establishing time-area closures, new technologies such as vessel monitoring and tracking systems (VMS) combined with continuous satellite tagging of EP turtles (for near–real time high-use data) could further mitigate human interactions with leatherbacks.

The implementation of time-area closures to protect leatherbacks in the South Pacific would provide parallel conservation benefits for other marine species [35–42] whose movements through the ETP region have also been revealed by satellite tracking and other data. For example, recent satellite tracking studies on green turtles (Chelonia mydas) from Galápagos have indicated that February–April closures to protect green turtles would also benefit migrating leatherbacks that use similar migratory and foraging habitats with the EP [42].

Large-scale electronic tagging studies will increasingly play an important role in informing spatio-temporal management activities of coastal and pelagic habitats for threatened marine species [5]. Our results elucidate the oceanic behaviors of leatherback turtles, and are applicable to existing and future conservation strategies that promote the recovery of EP leatherbacks (Box 1). In the future, animal movement models derived from satellite-tag data, in combination with real-time oceanography [20,32,43] will provide managers with the ability to predict the movement patterns of leatherback turtles and to take effective conservation actions to protect them at sea.

## Materials and Methods

### Tagging and data processing.

Leatherback sea turtles (*n* = 36) were instrumented with Sea Mammal Research Unit (SMRU) Satellite Relay Data Logger (SRDL) tags during 2004 (*n* = 17), 2005 (*n* = 8), and 2007 (*n* = 11). The SRDL tags were programmed to collect and transmit position, temperature, dive data, and tag diagnostic information [24]. We tagged ten additional turtles in 2004 with Wildlife Computer Smart Position Only (SPOT) tags, which were programmed to provide position data. We mounted the satellite transmitters on the turtles during oviposition using a harness technique [44] . Data from the tags were transmitted via the ARGOS satellite system [45].

We extracted tag-derived surface temperature measurements from the temperature-at-depth data transmitted by the SRDL tags. Surface was considered to be the first depth bin (mean = 5.1 m, sd = 0.7 m). A total of 5,787 temperature measurements were available after discarding 105 records because the first depth was missing, had a negative value, or had spurious position values.

### Track filtering and interpolation*.*

We generated final position estimates at regular 6-h intervals using state-space models (SSMs) [46,47] that were applied to the raw unfiltered satellite data to improve position accuracy and to align with SMRU summary dive data. The application of a switching SSM provided the capacity to discern between two behavioral modes based on a first-difference correlated random walk. The location of the switch between these two behavioral modes was used to objectively define the transition from inter-nesting (“mode 2”) to the post-nesting migration (“mode 1”) [47]. In cases where a clear switch was not present, we used a sudden change in the travel speed to determine the cut-off. For this paper, we only used the post-nesting portion of the tracks. Median daily speeds and headings were calculated from the interpolated tracks via first differencing consecutive points.

### Track current correction.

The observed track of an animal at any given time is the result of the animal's movement (swimming) plus the displacement caused by ocean currents (drift). The true behavior of a turtle can be thus obtained by removing the influence of currents on the animal's trajectory. We used surface current estimates obtained from the sum of the geostrophic and Ekman components, as measured by satellite [48], and removed them from the turtle movements at the locations generated with the SSMs. Within the equatorial band (4 °N–4 °S), a β-plane solution was applied [49].

### High-use area analysis.

We produced gridded utilization distribution maps [50] using a mesh size of 100 km^2^ and a fixed-kernel search radius of 0.5° for all years combined. The 95% utilization contour was used to define turtle high-use regions throughout the eastern tropical and South Pacific and the 75% contour to delineate the migration corridor between latitudes 12 °N and 5°S.

### Characterization of ocean currents.

We characterized the energetics of large-scale currents and their mesoscale fluctuations in the eastern tropical and South Pacific using merged satellite altimeter measurements of absolute dynamic topography and associated sea-level anomalies [51]. These data are generated by the Archiving, Validation, and Interpretation of Satellite Oceanographic data (Aviso) project at 1/3° resolution. Within five degrees of the equator, the Aviso product applies a β-plane solution [49] to obtain velocity and velocity anomaly vectors. We computed *MKE* from the mean *u*- and *v*- components of the geostrophic velocity as *MKE* = 0.5*(<*u*2> + <*v*2>). These calculations were performed separately for the February–April period of each tracking year, since the emphasis was on assessing the impact of inter-annual variability in geostrophic current strength on turtle migration while crossing the equatorial region. On the other hand, we computed *EKE* as a long-term mean for the period 14 October 1992–18 April 2007 from the mean geostrophic velocity anomalies (*u*′ and *v*′), as *EKE* = 0.5*(<*u′*2> + <*v′*2>). In this case, the emphasis was on examining turtle distribution in relation to a region of low mesoscale variability in the South Pacific Gyre.

### Phytoplankton CHL concentration.

The distribution of phytoplankton standing stock is a useful indicator of biogeography and ecosystem structure [24]. Near-surface CHL concentration, a proxy for phytoplankton standing stock, was obtained from Sea-viewing Wide Field-of-view Sensor (SeaWiFS) satellite ocean-color observations at 9-km resolution. We computed a long-term mean for the period September 1997–March 2007 for comparison of turtle movements in relation to phytoplanktonic biomass distribution throughout their range. Individual 8-d averages were also obtained for each turtle median daily position. The relationship between CHL and the turtles' median daily speed was investigated using linear regression, after log- and square-root-transformation, respectively, to meet normality assumptions.

### Digital bathymetry.

We extracted bathymetry from the global sea-floor topography of Smith and Sandwell [52], version 8.2 (November 2000) (http://topex.ucsd.edu/WWW_html/mar_topo.html). This dataset combines all available depth soundings with high-resolution marine gravity information provided by the Geosat, ERS-1/2, and TOPEX/Poseidon satellite altimeters, and has a nominal resolution of 2 arc min (∼4 km). The 2000-m isobath was extracted from this dataset to obtain the outline of the Cocos Ridge, the most prominent bathymetric feature in the migration corridor region (12 °N–5 °S) running northeast (∼43° azimuth) for ∼1,200 km between Galápagos and Central America.

### Geomagnetism.

Data on Earth's magnetic field (force and inclination) in the study area were calculated using the software GeoMag 6.0, available from the NOAA National Geophysical Data Center (http://www.ngdc.noaa.gov/seg/geom_util/geomutil.shtml), and the most recent (2005) International Geomagnetic Reference Field 10th generation (IGRF-10) coefficients.

## Supporting Information

Figure S1 Surface Currents and Vertical Thermal Structure in the Eastern Tropical and South Pacific Schematic representation of near-surface currents and vertical thermal structure in the eastern tropical and South Pacific, based on climatological annual data. (A) Current vectors (black) overlaid on current magnitude (colors; in cm s^−1^). Dashed black line denotes subsurface flow; dashed white line indicates a section along 95 °W.
(B) Surface zonal (black arrows) and meridional (orange arrows) velocities (in cm s^−1^) along 95 °W.
(C) Water-column temperature (colors; in °C) and the 15, 20, and 25 °C isotherms (black contours) along 95 °W. Zonal currents are represented as encircled x's for westward flows and encircled dots for eastward flows. Abbreviations are defined in the text.
(2.57 MB TIF)

Text S1 Currents and Thermal Structure of the Eastern Tropical and South Pacific (29 KB DOC)

## Abbreviations

CHL: chlorophyll-a
CRD: Costa Rica Dome
EKE: eddy kinetic energy
EP: eastern Pacific
ETPS: Eastern Tropical Pacific Seascape
EUC: Equatorial undercurrent
MPA: marine protected area
MKE: mean kinetic energy
PNMB: Parque Nacional Marino Las Baulas
SEC: South Equatorial Current
