# Supplementary material for: Persistent Leatherback Turtle Migrations Present Opportunities for Conservation
Source: PLoS Biol. 2008 Jul 15;6(7):e171. doi: 10.1371/journal.pbio.0060171 (PMC2459209; doi:10.1371/journal.pbio.0060171)
Supplement: Supplementary file 1 [file pbio.0060171.sd001.doc]

**Text S1**

**Currents and thermal structure of the eastern tropical and South Pacific**

Absolute geostrophic velocity data from Aviso was used to produce a long-term (14 October 1992 - 18 April 2007) representation of near-surface currents in the study area. For in-depth information about these currents the reader is referred to a recent review [1]. Briefly, from south to north (Figure S1A), they include: the Peru-Chile Current System (PCCS) flowing northward along the coast of South America; the westward South Equatorial Current, with its southern and northern branches on either side of the equator (centered at about 4°S and 3°N, respectively); the eastward Equatorial Undercurrent (EUC) straddling the equator between 1°N and 2°S; the eastward North Equatorial Countercurrent (NECC), which seasonally extends from the central into the eastern tropical Pacific (centered near 6°N at longitude 95°W; Figure S1B); and, finally, the cyclonic circulation associated with the Costa Rica Dome (CRD) centered at 9°N, 90°W, whose return (westward) flow to the north forms the Costa Rica Coastal Current (CRCC). It is noted that while the EUC is a strong eastward flow in excess of 50 cm s^-1^ at 100-m depth, this subsurface current is only evident as a decrease in westward speed (or slightly eastward speed at 2°S) in the altimeter-derived data (Figure S1B).

Climatological annual temperature data from the World Ocean Atlas 2005 [2] (WOA05) was used to examine the vertical thermal structure of the study area along 95°W (Figure S1C). The dominant feature is the series of meridional ridges and troughs in the thermocline (contained within the 15, 20, and 25°C isotherms) that are driven by variations in regional wind-stress curl. The resulting pressure gradients give rise to the system of equatorial currents described above. The thermocline ridge centered at about 9°N is associated with the NECC-CRD complex, while outcropping of the 25°C isotherm just north of the equator marks the location of the Equatorial Front (EF) (Figure S1C).

**References**

1. Kessler WS (2006) The circulation of the eastern tropical Pacific, A Review. *Progr. Oceanogr*. 69: 181-217.
2. Locarnini RA, Mishonov AV, Antonov JI, Boyer TP, Garcia HE, (2006) In: Levitus S, editor. *World Ocean Atlas 2005* Washington, D.C.: U.S. Government Printing Office (NOAA Atlas NESDIS 61).

**Figure S1.** Surface currents and vertical thermal structure in the eastern tropical and South Pacific

Schematic representation of near-surface currents and vertical thermal structure in the eastern tropical and South Pacific, based on climatological annual data. (A) Current vectors (black) overlaid on current magnitude (colors; in cm s^-1^). Dashed black line denotes subsurface flow; dashed white line indicates a section along 95°W.

(B) Surface zonal (black arrows) and meridional (orange arrows) velocities (in cm s^-1^) along 95°W.

(C) Water-column temperature (colors; in °C) and the 15, 20, and 25°C isotherms (black contours) along 95°W. Zonal currents are represented as encircled ‘x’s for westward flows and encircled dots for eastward flows. Abbreviations are defined in the text.
